# Who Was Helping? The Scope for Female Cooperative Breeding in Early *Homo*

**DOI:** 10.1371/journal.pone.0083667

**Published:** 2013-12-18

**Authors:** Adrian Viliami Bell, Katie Hinde, Lesley Newson

**Affiliations:** 1 Department of Anthropology, University of Utah, Salt Lake City, Utah, United States of America; 2 Department of Human Evolutionary Biology, Harvard University, Cambridge, Massachussetts, United States of America; 3 Brain, Mind, and Behavior Unit, California National Primate Research Center, University of California Davis, Davis, California, United States of America; 4 Department of Environmental Science and Policy, University of California Davis, Davis, California, United States of America; 5 College of Life and Environmental Sciences, University of Exeter, Exeter, United Kingdom; Durham University, United Kingdom

## Abstract

Derived aspects of our human life history, such as short interbirth intervals and altricial newborns, have been attributed to male provisioning of nutrient-rich meat within monogamous relationships. However, many primatologists and anthropologists have questioned the relative importance of pair-bonding and biparental care, pointing to evidence that cooperative breeding better characterizes human reproductive and child-care relationships. We present a mathematical model with empirically-informed parameter ranges showing that natural selection favors cooperation among mothers over a wide range of conditions. In contrast, our analysis provides a far more narrow range of support for selection favoring male coalition-based monogamy over more promiscuous independent males, suggesting that provisioning within monogamous relationships may fall short of explaining the evolution of *Homo* life history. Rather, broader cooperative networks within and between the sexes provide the primary basis for our unique life history.

## Introduction

Human life history is puzzling. Despite producing relatively large, altricial newborns that grow expensive tissues (e.g. our brain) [1], [2], we reproduce at a faster rate than our closest primate relatives [3]. This is possible because human mothers receive a considerable amount of help caring for and provisioning their young [4] with substantial variation in what help is provided to mother and who provides it. Since females of all other members of the of the Homininae sub-family raise their young without help, shared parenting likely emerged in the hominine line since the split with the last common ancestor of chimpanzees, bonobos and humans.

However, some have attributed this shift to male provisioning in monogamous relationships. The lack of sexual dimorphism in the bipedal ape *Ardipithecus radius*, a human ancestor who lived 4.4 million years ago, suggests to some that males and females pair-bonded, making monogamy and biparental care the ancestral condition in earlier *Homo* [5]. Thus “Man the Hunter” helped produce higher quality offspring at a faster rate by provisioning his pair-mate and her offspring with meat. This has led to the development of a substantial literature around how individuals select, attract, and monitor mates based on the assumption that selection favored adaptations integral to monogamous pair-bonds and bi-parental infant care, e.g. [6], [7].

Yet empirical studies suggest that humans have evolved to engage in broadly cooperative food-sharing and infant care networks [4]. All human mothers receive help provisioning and caring for their children from many people, not just the infant’s father [4], [8], [9]. Male provisioning to putative offspring varies substantially across habitats and cultural groups [8], [10], and the death or absence of a father has been found to have no effect on child survival in some horticultural and hunter-gatherer subsistence populations [9], [11]. This outcome is likely due to the fact that infants and children receive care from a network of individuals that can include grandparents, aunts, uncles, siblings, distantly-related kin, and non-kin, a reproductive strategy more consistent with cooperative or communal breeding [4], [12]–[14]. Here, the sharing and caring derived from the pooled energy of such a network is an integral component of human life history [15].

**Figure 1:**
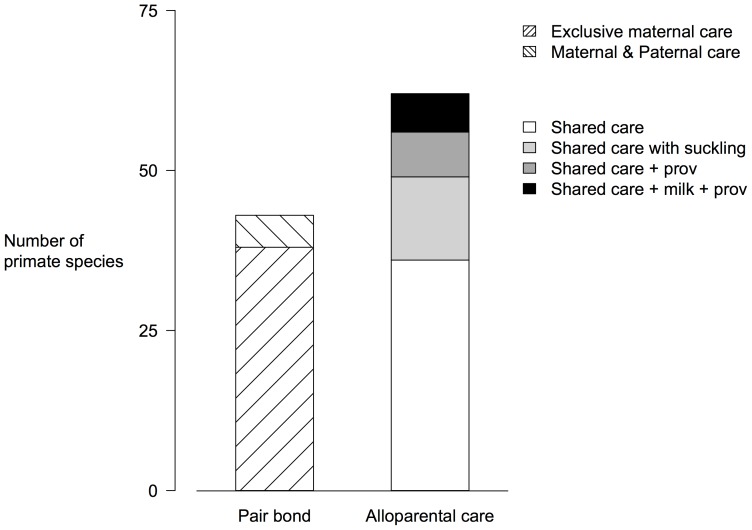
Infant care classification for 105 primate species, from the appendix to Sarah Hrdy’s treatise on infant care [4], [52]. Hrdy’s classification [52] follows: *Exclusive maternal care*: mother is very possessive and is the only one to hold and carry her infant. *Maternal and paternal care*: mother allows male she is paired with to take and carry infant and he is eager to do so. In New World monkeys, infant may actually take the initiative in transferring to “father.” Typically, the mother’s mate is the main caretaker, and alloparents are rarely involved. *Shared care*: mother is tolerant and allows allomothers to take and carry her infant within 3 weeks of birth. *Shared care with suckling*: group members other than the mother care for infants, and if the allomother is lactating, she allows an infant other than her own to suckle. Allomaternal suckling may range from occasional and brief access to more sustained access, as in species where two mothers share a nest. *Shared care + prov*: provisioning ranges from minimal to extensive. *Shared care + milk + provisioning*: combinations of behaviors described above.

Females from a broad range of mammalian taxa often directly or indirectly care for offspring that are not their own (Figure 1). Among wild-living white headed capuchins (*Cebus capucinus*), females regularly nurse one another’s infants, with a higher rate of allo-nursing among low-ranking mothers [16]. Allo-nursing in tufted capuchins (*Cebus nigritus*) has been hypothesized to serve significant social benefits [17]. Female chacma baboons (*Papio hamadryas ursinus*) form stable, close, persistent social bonds known to enhance fitness, despite not engaging in cooperative infant care [18]. Among maternal relatives in the mouse lemur (*Microcebus murinus*), mothers recognize their own infants but will allow other female’s infants to suckle in communal sleeping nests [19]. Banded mongoose females (*Mungos mungo*) tradeoff protective babysitting roles and often give birth synchronously, which maximizes pup survival by reducing asymmetry in pup competitiveness and inhibiting infanticide [20]. Here, forgoing cooperation by evicting subordinate “babysitter” females appears costly because dominant females gain less mass during pregnancy, fewer of their pups survive, and the pups that do survive to independence are smaller [21]. Among house mice (*Mus domesticus*), pairs of females cooperatively nurse each other’s pups and defend the shared nest. These cooperative relationships between house mice females are not necessarily contingent on relatedness, are established before conception, and can persist across reproductive events [22]. Most importantly females show partner preference which produces more egalitarian relationships and increases reproductive success [23].

When males or coalitions of males contribute parental effort it may be a byproduct of actions that could be classified as mating effort or efforts to build or maintain coalitions. For example, male chimpanzees engage in cooperative territorial defense [24], mate-guarding [25] and hunting [26], and they have been observed to share meat with allies [27] and estrous females [28]. This may contribute to the survival of females and their young but does not constitute direct paternal care [29]. In humans, successful hunters do not just provision their pair-mate and offspring; they cooperate with and share their gains with the wider local community [10], [30], [31]. A recent phylogenetic analysis across primates demonstrates that the capacity to share food among adults evolved in the context of exchanges of coalitionary support or mating opportunities [32]. Although notably, adult food sharing has only occurred in taxa where food sharing with infants was also present.

The cohesive picture that emerges from these empirical studies is that humans have evolved to engage in broadly cooperative food-sharing and infant care networks, rather different from the behavior observed in strongly pair-bonded primate species such as gibbons and siamangs [33]. To explore the scope for the emergence of cooperative relationships in child care, we formulate and analyze a mathematical model. While similar in purpose to [7], in contrast our model includes cooperation between females and cooperation between males. We have envisaged a precise mechanism with hypothesized parameters ranges to test the robustness of a particular childcare strategy. Since some parameters considered here have not received significant research effort (i.e. allo-maternal lactation), our model provides motivation for further empirical investigations.

In the model we consider three female and two male provisioning and reproductive strategies. The fitness of each strategy is determined by the infant care roles played between females, between males, and between females and males. One primary goal of the model is to relate its formulation to the empirical knowledge of primate behavior and life history. Therefore the model is developed to describe the reciprocal behavioral interactions among females and males. We assume the benefits of cooperation between females to be improved foraging efficiency of females when not encumbered by infants such as has been observed in some other primates [34]. This will lead to increased infant survival and decreased interbirth interval. In contemporary humans decreased interbirth interval is achieved by “complementary feeding”, i.e. provisioning of unweaned infants with foraged food. We assume that this practice evolved after the establishment of cooperative breeding but that reduction of interbirth interval can be achieved if mothers nurse each others young.

### Female and Male Strategies

For females, we consider three possible reproductive strategies: (*a*) an Independent Mother, (IM) who strictly provisions her own children, (*b*) a Cooperative Mother (CM), who engages in allo-parenting with another CM, and (*c*) a Opportunistic Mother (OM), who accepts allo-parenting benefits but does not reciprocate by being an allo-parent. Let the two strategies available to males be a Coalition Male and a Non-coalition Male. Non-coalition males forage independently and thus gains fewer resources for paternal care than those in coalitions, but they are able to engage in more matings than males in coalitions. A Coalition Male will form a coalition with another Coalition Male with whom he will not compete for mates. Coalition Males can invest more effort in parental care because (1) they reap an economy of scale and (2) they also expend less effort competing for mates and mating [31]. Coalition males engage in fewer matings than Non-coalition Males due to enhanced cooperation and mutual monitoring between males.

To calculate the fitness of the male and female strategies we account for all possible interactions in which males and females may engage in a large population. Female strategies pair at random with male strategies, though CMs pair with Coalition Males with probability 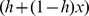, where 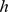 is the strength of positive assortment for male-female cooperative strategies and 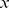 is the frequency of Coalition Males. Coalition Males will either be in a coalition with probability 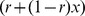 or not with probability 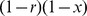, where 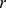 is the kin selection parameter. Larger values of 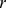 give a greater likelihood of like-strategies to interact. Similarly, Non-coalition Males assort with their own strategy with probability 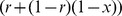 and with Coalition Males with probability 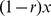. Similarly, females also interact with CM, IM, and OM mothers depending on their frequency in the population and the strength of kin selection. For example, with 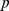 as the frequency of Cooperative Mothers, two partnered CMs occur in the population with probability 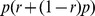.

## Results

### Evolution of Cooperative Mothers

**Figure 2:**
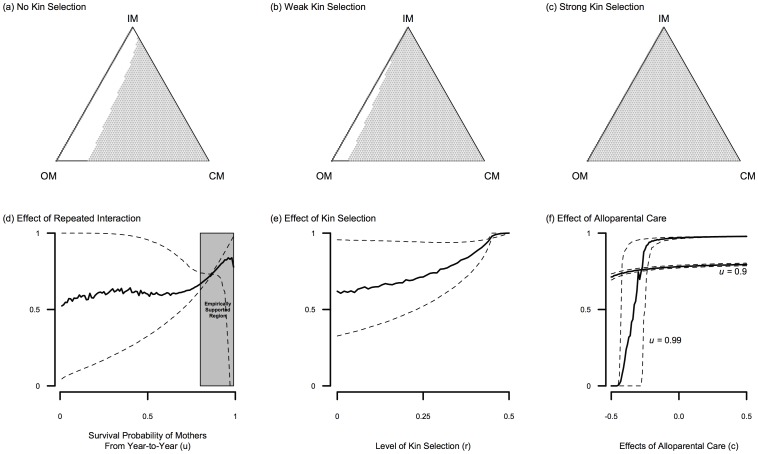
Model analysis illustrating the scope for cooperative mothers. The upper row describes a deterministic process of the evolutionary dynamics of the three female strategies: Independent Mother (IM), Opportunistic Mother (OM), and Cooperative Mother (CM). The gray region is when selection favors CM, white region is when OM is favored, and the thicker dark line is where the fitness of OMs and IMs are the same. Panels (a)–(c) assume parameter values 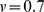, 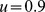, 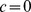, 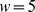, 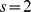, 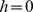, 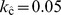 and 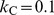. However panel (a) assumes no kin selection (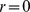) and panel (b) prescribes weak kin selection (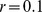), and panel (c) specifies strong kin selection (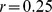). The bottom row of panels describes the basin of attraction for Cooperative Mothers through stochastic simulation as a function of the repeated interaction parameter 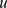 (panel (d)), level of kin selection (panel (e)), and the effect of alloparental care (panel (f)). The position of the unstable equilibrium between OM and CM females shown in the ternary plots above defines the basin of attraction. The dashed curves are 95% confidence bounds around the mean (solid line) computed by taking 1000 random uniform parameter values within the ranges reported in Table 1 for each value of 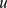, 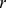, and 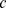 on the horizontal axis for panels (d)–(f), respectively.

Our analysis shows a very large scope for the evolution of Cooperative Mothers. This is because the basin of attraction for CMs remains large over hypothesized parameter values (Table 1) and small amounts of kin selection makes CM the dominant strategy. Figure 2(a) shows that density-dependent dynamics between the three strategies heavily favors CMs under certain parameter values, with Figure 2(b–c) showing that small amounts of assortment through kin selection can make CMs favored at any frequency. Monte Carlo simulations via drawing uniformly from the parameter in Table 1, show that this basin of attraction remains in favor of CMs even at low survival probabilities (Figure 2(c)). The wide confidence intervals in Figure 2(d) at low survival probabilities (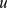) reflect highly variable “search costs” for a CM to find another CM – half the time the CM pays the cost of partner defection, and the other half behaves as an IM. Further, since OM is the only strategy receiving significant alloparental care at these specifications, OMs suffer fitness costs when alloparental effects are detrimental (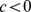), leaving CMs to be favored.

**Table 1:** Description of parameters in the evolutionary model and the ranges used in the Monte Carlo simulations.

Parameter	Description	Hypothesized range	References/Justification
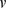	baseline survival probability ofoffspring to a breeding adult	0.5−0.75	In extant *Pan*, survival to reproductive maturity is 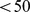 % but is much higher in hunter-gatherer human populations; we therefore randomized mortality risk between chimpanzee and modern human parameters [36].
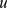	survival probability of a motherfrom one year to another	0.90−0.99	Among baboons, chimpanzees, and hunter-gatherer human populations the annual mortality rate for adult individuals ranges from 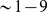 % [35], [36], [53], [54].
**Effects of the female reproductive strategy**			
	marginal change in offspring survivalto adulthood (as a breeding adult)as a result of allo-parenting	−0.5−0.5	No direct study measures the effects of allo-parenting on offspring survival to adulthood, therefore the range represents both extreme net costs and net benefits. However, this is a conservative parameter range as there is evidence that allo-parenting likely yields net benefits to weaning age (mongooses; house mice) and particularly when allo-parents are closely related [19].
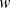	interbirth interval (IBI) for independentmothers who do not share the “lactationalload” through allo-maternal nursingand allo-caring	5 years	This assumes an ancestral IBI more reminiscent of extant chimpanzees, 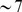 years following the production of a surviving offspring [55].
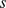	decrease in IBI for mothers who share the“lactational load” through allo-maternalnursing and allo-caring	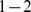 years	Lengthening inter-nursing intervals for foraging while another mother cares and possibly nurses one’s infant, is expected to down-regulate milk synthesis, alter hormonal regulation, and subsequently shorten IBI [47].
**Effects of the male reproductive strategy**			
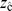	extent of lifetime extra-pair matings(EPM) by Non-coalition Males	0−0.2 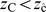	Independent males engage in EPM more often than coalitionary males. We hypothesize that from a chimpanzee model of males sharing mating opportunities with allies [28], human “respect” for coalition partners’ mating relationships may have emerged. As a result coalition males would have greater probability of exclusive mating relationships with females but less opportunity for EPM among vigilant coalitionary partners. A conservative measure of fathers’ benefits to offspring survival. Father’s effect on infant survival is variable across cultures but is generally low [9]. Assumes fathers in coalitions experience an economy of scale in hunting returns and provide greater benefits to offspring survival despite distributing food broadly throughout the group [31].
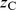	extent of lifetime EPM by aCoalition Male	0−0.05 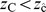	
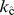	marginal change in the probability tosurvival to a breeding age as a result ofpaternal care by a Non-coalition Male	0−0.05 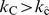	
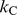	marginal change in the probability tosurvival to breeding age as a result ofpaternal care by a Coalition Male	0−0.1 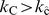	

The simulations drew values uniformly across these parameter ranges.

With higher survival probabilities the scope for CMs becomes even higher as the longer time horizon of allo-mothering can better offset the costs to defection by dishonest mothers. Empirical work in baboons, chimpanzees, and humans [35], [36] suggests low adult female mortality rates, which would likely extend the time horizon long enough for cooperation to pay-off. So despite the fact that both Cooperative Mothering and “dishonest” Opportunistic Mothering are evolutionary stable strategies in some conditions, the probability of Cooperative Mothers successfully invading and remaining stable is very high (Figure 2).

Under some parameter sets where the survival probability is high (e.g. 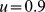), the effects of alloparental care (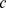) are either very slight or extremely key to selection favoring CMs. With extremely high survival probabilities (e.g. 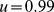) the scope for CMs becomes more uncertain (Figure 2(d)) precisely because strong negative alloparental effects (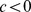) over very long time horizons will drastically decrease CM fitness. However, in this model, a less detrimental, neutral, and positive value of alloparental care, under the same time horizon, will strongly favor CMs (Figure 2(f)).

These results highlight the importance of search time and the cooperative time horizon in this model. Figures 2(b)–(c) show that even a little kin selection (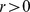) has a large positive effect toward CM evolution. This is because kin selection decreases the search time for CMs to pair, interact and reap the rewards of decreased birth intervals through allo-mothering. (Note that Figure 2(d) uses very conservative parameter values on CM evolution as 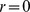 and the frequency of IMs is zero.).

In sum, our conservative analysis is suggestive of the possible role of Cooperative Mothers early in hominid evolution. A larger fraction of Independent “honest” Mothers would further increase the scope for CMs. Would higher frequencies of Coalition Males also promote Cooperative Mothers and vice versa? After we analyze the model’s support (or not) for the evolution of Coalition Males, we look for any synergistic dynamic between allo-mothering and coalition-based monogamous male foragers.

### Evolution of Male Coalitions and Monogamy

**Figure 3:**
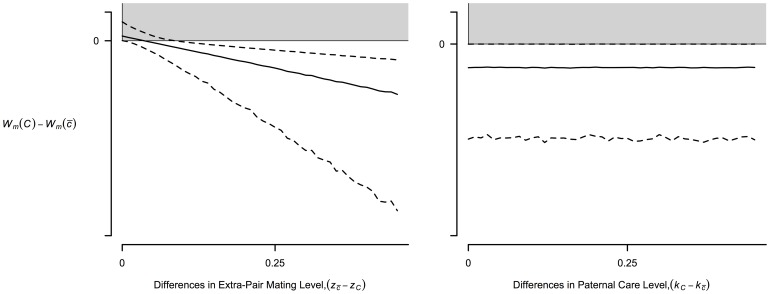
Plot of the fitness differences between Coalition Males (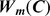) and Non-coalition Males (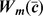) as a function of the differences in the level of Extra-Pair Matings (EPMs) and childcare. For the left panel the level of EPMs for Coalition Males is set at 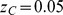. The right panel has the level of paternal care for Non-coalition Males set at 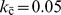. The solid line with its respective confidence intervals (dashed lines) were estimated through simulation by drawing 10,000 random parameter sets from Table 1. Gray regions highlight when Coalition Males are favored, with the corresponding white region showing when Non-coalition Males are favored. The level of kin selection (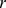) and positive assortment between Cooperative Mothers and Coalition Males (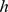) is zero.

Analysis through Monte Carlo simulation shows mixed support for Male Coalitions to evolve. As in a recent work [7], we find extra-pair matings (EPMs) by Non-coalition Males to be the primary obstacle for Coalition Males. The gray regions in Figure 3 show when draws from the parameter ranges in Table 1 results in a fitness advantage for Coalition Males on average (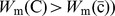). As Non-coalition Males increase in EPMs, it becomes even more difficult for Coalition Males to evolve. Increased paternal care by Coalition Males has little effect because of the “push-pull” dynamic of Coalition Males increasing their reproductive success while simultaneously increasing the benefits to EPMs (Figure 3, c.f. [37]).

**Figure 4:**
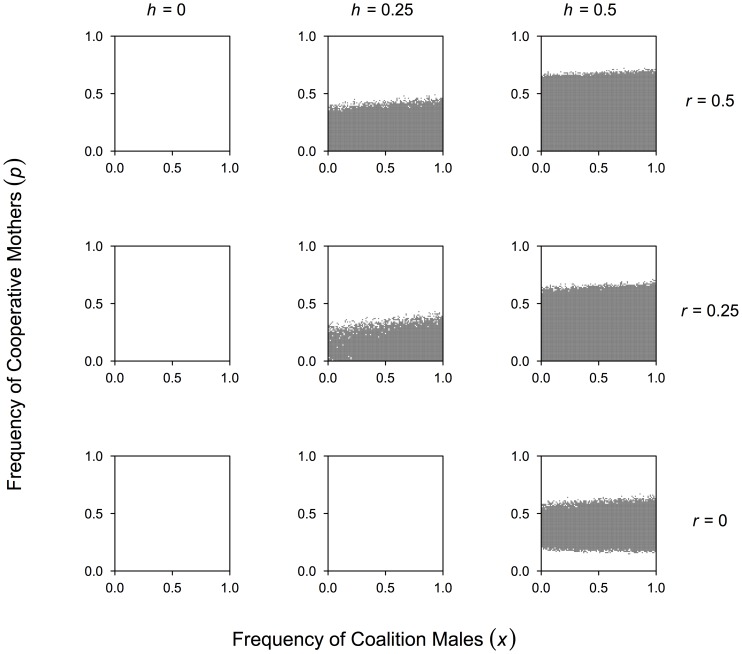
Kin selection, male-female cooperative assortment and density-dependent evolutionary dynamics of Coalition Males. For all panels, each frequency of Cooperative Mothers (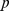) and Coalition Males (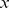), 100 parameter sets were randomly drawn from ranges in Table 1 to estimate the distribution of when the fitness of Coalition Males is greater than that of Non-coalition Males (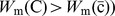). Dark regions indicate when the mean of those distributions at a specific value of 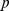 and 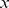 favor Coalition Males. The level of kin selection (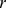) is the same across panel rows and the level of positive assortment between Cooperative Mothers and Coalition Males (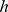) is the same within a column of panels.

However, positive assortment of Cooperative Mothers and Coalition Males can generally help favor Coalition Males. Figure 4 shows that kin selection alone, which produces more frequent cooperative pairs of females and pairs of males, does not increase the fitness advantage to Coalition Males unless cooperative males and females also assort positively. Once this occurs (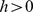), then kin selection within the sexes can expand the scope for Coalition Males.

Some patterns in Figure 4 provide further insights. In all cases explored here, when Cooperative Mothers are very common Coalition Males are not favored. This is because the average fitness of females increases and Non-coalition males that engage in more EPMs surpass the fitness of Coalition Males. At lower levels of kin selection and with some male-female cooperative assortment (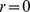 and 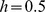 in Figure 4), there are lesser benefits to pairing with CMs, and Non-coalition Males who interact with the more common IMs and OMs with higher EPMs have a fitness advantage. Once higher levels of kin selection are reached (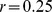), this advantage to Non-coalition males is erased.

This analysis highlights the importance of the parameter ranges which specify the level of extra-pair matings for Coalition and Non-coalition males and whether there exists any assortment within and between the sexes. Given our current hypothesized parameter ranges in Table 1, we find a fairly restricted range under which a male coalition-based monogamy can evolve without the non-random association of female cooperative breeders.

## Discussion

Our analysis suggests that female cooperation, rather than bi-parental care, would have provided a more robust system for helping hominid mothers care for infants. Consistent with recent work [7], we find male provisioning of a single female and her infant unlikely to be evolutionarily stable. In contrast, however, [7] finds that biparental care could evolve if females can choose to mate only with one male and “faithful” females mate with males who provision them and their joint offspring. The primary difficulty here is whether females can “choose” to be faithful. Exerting choice would require the female being able to protect herself and her infant from other males while her pair-bonded mate is away foraging. This can be envisaged in species where females are larger than males but the fossil evidence reveals that in members of the Homo and Australopithicine genus, males were generally larger, sometimes considerably larger than females [38]. Even in monogamous non-sexually dimorphic primates, like gibbons and titi monkeys, the males do not leave their mate and offspring to forage and bring back food. If early Homo males did provide parental care, it might have taken the form seen in titi monkeys in which the male accompanies the female while she forages carrying their infant, handing it to her to suckle [39], [40]. By carrying the infant, a male can reduce energetic demands on the female while protecting her from other males and thus maintaining paternity certainty.

The monogamy observed in modern humans may have evolved in a context of multi-male, multi-female cooperative breeding group. As groups evolved increasingly complex culture, social recognition of long term relationships between mates may have emerged through a process of gene-cultural coevolution [41], [42], rather than through the intra-sexual aggression that can de facto maintain monogamy in other species, such as titi monkeys and gibbons [33]. Indeed a recent phylogenetic analysis of primate social organization reveals that monogamy derives from earlier multi-male, multi-female social groups [43].

It is argued that females cooperating in the care of young is highly unlikely because of low relatedness between females who disperse on maturity [7]. Our model shows, however, that high levels of relatedness are not necessary to establish cooperation between females. A little kin selection accelerates cooperation but cooperative strategies are not contingent on kin selection. Cooperative mothers are favored even when relatedness is relaxed in the model (Figure 2(a)). This is a particularly compelling result given that none of our closest living relatives are characterized by female philopatry, so opportunities to cooperate among adult female relatives are likely to have been limited. However evidence from chimpanzees suggests that unrelated adult females can form persistent dyadic social bonds [44]. During hominid evolution, natural selection may have favored and expanded such ancestral social bonds into the cooperative networks we find in humans today.

For cooperative mothering to be a compelling strategy, a division of labor between mothers would have to generate sufficient extra resources to allow a reduction in interbirth interval. The lack of cooperation seen among female apes suggests that it is not sufficiently beneficial in their case. However, if Australopithecine females produced larger babies than extant apes, as [1] suggests, the costs to mothers of constantly carrying infants would be greater and so the benefits of taking turns to babysit each others infants would be greater. Creching infants would be more beneficial still in habitats in which resources are less evenly distributed, forcing foragers to walk longer distances without access to water. Fossil evidence suggests that by the time of the emergence of early *Homo*, hominids occupied the drier, less stable and more heterogeneous environments that were expanding in Africa during the early Plio-Pleistocene [45], [46]. In these habitats, a division of labor which allowed mothers to spend time foraging unencumbered by an unweaned infant would have made it possible for them to gain the extra resources necessary to provide allomaternal, as well as maternal care. Moreover, recent work suggests that human breast-milk is slightly more energetically dense than is the milk of great apes, which may be due in part to longer inter-nursing intervals [47]. It is difficult to imagine reciprocal infant care-taking among mothers that does not include allo-maternal nursing to ameliorate infant hunger and fussiness. Allo-maternal nursing is more common across human cultures than is generally appreciated. Within Islamic culture, there is the practice of “milk kinship.” In such instances infants are nursed by a woman not their mother, and consider her biological children “milk brothers” and “milk sisters” [48]. Allo-maternal lactation has been reported to be routine among the Efe, Aka, Ongee, Beng, and Trobriand Islanders [4]. Although less studied, cross-nursing occurs among mothers in modern Western societies [49].

## Conclusion

By the time members of the hominid line began to exploit drier and more varied habitats about two million years ago, it is likely they lived in cooperative breeding groups. If this is the case, then speculation about the course of human evolution and the adaptations thought to have emerged ought to consider what phenotypes would have been favored in this social system. Cooperative breeding groups would not have provided the selective environment favoring individuals with a capacity for choosing mates, nor individuals with a inclination to form a long-term attachment to a mate, nor males with an inclination to provide certain kinds of parental care (e.g. [6]). Rather, perhaps as in some contemporary cultures [50], [51], the matching up of reproductive age people was done cooperatively with friends and relatives playing a role in creating the match. Current human reproduction relies on cultural mechanisms that ensure children receive adequate care. Institutions such as marriage formalize family responsibilities, social norms control timing of births and facilitate cooperation and coordination of parenting effort, and tools and technology help to protect and confine infants and aid in the production of safe weaning foods.

Our mathematical analysis suggests that female cooperatives are foundational to our modern life history. Nevertheless, our results should be taken as preliminary as work is needed to consider other potentially important factors and address further the parameter ranges given in Table 1. For example, one vital question is whether there is a positive correlation between male coalitions that produce an economy of scale and paternity certainty. Moreover, males may benefit from cooperation with females if females often target less variable food items. Model development and empirical work may also elaborate on the relationship between the costs and benefits of allo-parenting between cooperative kin and non-kin. Positive assortment between cooperative males and females through female choice, for example, may expand the scope for Coalition Males. According to our current estimation, however, male coalition-based monogamy is likely to have evolved secondarily to a primary cooperative basis in infant care established by mothers.

## Methods

### Maternal Interactions and Reproductive Success

Reproductive payoffs to females are described below, with the mathematical details in Information S1.

#### Cooperative mothers

A Cooperative Mother may interact with another CM, Independent Mothers (IM), or an Opportunistic Mother (OM). After CMs interact with an IM or OM once, they will search for another female partner. This continues until the CM meets another CM, then the two engage in cooperative allo-parenting *ad infinitum*. Thus the fitness of CMs is divided into interactions with non-cooperative and cooperative female partners with reproductive payoffs discounted by the probability of the interaction taking place.

When two CMs interact each has an equal probability of being the allomother first, engages in alloparental care, then enters in reciprocal interactions in the next year with probability 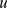, where 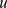 is the probability of a mother’s survival from year-to-year. This continues *ad infinitum*. The survival probability of offspring to the breeding adult stage, without allo-parental and paternal care, is 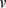, the interbirth interval of Independent Mothers is 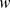 and the shortening of the interbirth interval due to allo-maternal care is 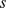.

When a CM interacts with an OM there is a 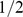 chance the OM will reproduce first and gain the benefit of the allo-parent without reciprocating. The other 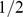 chance the CM will opt to reproduce first but the OM will refuse to allo-parent, and two revert to acting as IMs. Thus, half the time a CM interacting with an OM receives the “suckers” payoff, because the CM paid costs as an allo-mother (delaying reproduction) without reciprocated benefits from the OM.

#### Opportunistic and independent mothers

When OMs interact with an IM or another OM they both act as independent caregivers. An Independent Mother’s reproductive payoff is not contingent on any interactions with other females.

Female and male reproductive output are tied together since offspring receive paternal care of amount 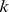 and males gain a certain number of offspring conditional on the female strategy. Below we specify the male strategies and their reproductive outcomes.

### Paternal Interactions and Reproductive Success

Males can provision (or not) his offspring by a female with one of the strategies described above. Males who form coalitions and reap an economy of scale will have different fitness consequences due to a likely increased support of their children and less extra-pair reproductive activity. The paternal contribution to infant care, 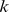, takes on the value 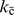 when females pair with an male who forages independently of other males and 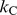 when females pairs with a male who forms coalitions with other males. Since males in coalitions gain more per capita resources, then .

Extra-pair reproductive activity for Coalition Males is 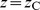 and for Non-coalition M ales is 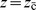, where 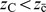. With 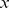 as the frequency of Coalition Males, let 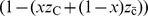 be the probability that a male’s provisioning goes toward his own biological offspring, indicating that an increase in extra-pair reproductive activity decreases paternity certainty in the population. Paternity certainty increases with a higher frequency of Coalition Males. For simplicity we assume that males engaging in extra-pair reproduction have equal access to all females, thus the fitness gain for extra-pair reproduction throughout a male’s lifetime is 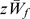, where 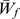 is the mean fitness of females.

## Supporting Information

Information S1 **Derives fitness expressions for the female and male strategies described in the main text.**
(PDF)
