# Correction: Who Was Helping? The Scope for Female Cooperative Breeding in Early *Homo*

**DOI:** 10.1371/annotation/c62b2301-19ce-4ed5-85a3-123e8d213030

**Published:** 2014-01-02

**Authors:** Adrian Viliami Bell, Katie Hinde, Lesley Newson

In the second paragraph of the Introduction, "*Ardipithecus radius*" should instead read "*Ardipithecus ramidus*."

