# Supplementary material for: Who Was Helping? The Scope for Female Cooperative Breeding in Early Homo
Source: PLoS One. 2013 Dec 18;8(12):e83667. doi: 10.1371/journal.pone.0083667 (PMC3867437; doi:10.1371/journal.pone.0083667)
Supplement: Information S1 — Derives fitness expressions for the female and male strategies described in the main text. (PDF) [file pone.0083667.s001.pdf]

Supporting Information: Who was Helping? The Scope for Female Cooperative Breeding in Early *Homo*

Adrian V. Bell [1], Katie Hinde [2,3], and Lesley Newson [4,5]

[1]Department of Anthropology, University of Utah
[2]Department of Human Evolutionary Biology, Harvard University
[3]Brain, Mind, and Behavior Unit, California National Primate Research Center, University of California, Davis, USA
[4]Department of Environmental Science and Policy, University of California, Davis, USA
[5]College of Life and Environmental Sciences, University of Exeter, United Kingdom

November 22, 2013

1 Calculation of fitness expressions

A mother may participate in several different types of interactions with other mothers.

1.1 Cooperative Mothers

Cooperative Mothers (CMs) may interact with their own type, Independent Mothers (IMs), or Opportunistic Mothers (OMs). When CMs interact with an IM or OM once, they will search for another female partner. This continues until the CM meets another CM, then the two engage in cooperative allo-parenting *ad infinitum*. Thus the fitness of CMs is divided into interactions with non-cooperative and cooperative female partners with reproductive payoffs discounted by the probability of the interaction taking place. Let t_{NC} be the expected time until the next birth during the time when CMs interact with IMs or OMs. Thus,

$$t_{NC} = \frac{q}{1-p^*}w + \frac{1-p^*-q}{1-p^*}\frac{1}{2}\left(w + (1+w)\right) \tag{1}$$

where w is the interbirth interval without allo-mothering, and $1+w$ the interbirth interval when a CM alloparents a OM's offspring without the OM reciprocating (delayed reproduc-

tion). Parameter p is the frequency of CMs, q the frequency of IMs, $1-p-q$ the frequency of OMs, and $p^* = r + (1-r)p$ is the kin-selection-adjusted probability of meeting another CM where r is the kin selection parameter. The number of interactions before a CM meets another CM can be specified as a random variable distributed as a Negative Binomial, yielding the mean number of interactions $\frac{1-p^*}{p^*}$. Therefore, for $\frac{1-p^*}{p^*}$ interactions, CMs will interact with IMs or OMs with probability $\frac{q}{1-p^*}$ and $\frac{1-p^*-q}{1-p*}$, respectively, gaining the expected reproductive payoff of V_{CIO}. The reproductive payoff to interacting with another CM, V_{CC}, is discounted by the probability of the interaction starting (u^{t_C}). The time interval until a CM strategy is successful at meeting another CM strategy is,

$$t_C = t_{NC}\frac{1-p^*}{p^*} \tag{2}$$

Since u is the survival probability of each mother from year to year, u^{t_C} is the probability of a CM surviving to begin interacting with another CM. Put together, the fitness of the CM strategy becomes,

$$W_{CM} = u^{t_C}V_{CC} + V_{CIO}\sum_{i=1}^{\frac{1-p^*}{p^*}} u^{it_{NC}} \tag{3}$$

where V_{CC} and V_{CIO} is the expected reproductive payoff for a CM to interact *ad infinitum* with another CM, and a CM to interact with IMs and OMs, respectively.

Reproductive payoffs. When two CMs interact they continue their interaction in perpetuity. In the beginning, each has an equal probability of being the allomother first, engages in alloparental care, then enters in reciprocal interactions in the next year with probability u. The survival probability of offspring to the breeding adult stage, without allo-parental and paternal care, is v and s is the shortening of the interbirth interval due to allo-maternal care. As they trade child-bearing and allo-parenting duties, the expected time to the ith birth is,

$$t_{cb} = \frac{1}{2}\left(i(w-s) + (1 + i(w-s))\right) \tag{4}$$

and the reproductive payoff becomes,

$$V_{CC} = v(1+k+c)\sum_{i=0}^{\infty} u^{t_{cb}} = v(1+k+c)\frac{u^{1/2}}{1-u^{w-s}} \tag{5}$$

where vk and vc is the change in the survival probability of the offspring due to paternal investment and allo-mothering, respectively. The realized value of k depends on the male provisioning strategy, discussed below. Thus the quantity $v(1+c+k)$ is the total survival probability of an offspring receiving both paternal and allo-maternal care. We assume that this value does not exceed unity, thus our analysis assumes $\min(1, v(1+c+k))$.

The reproductive payoff for each $\frac{1-p^*}{p^*}$ interactions with the two non-cooperative mother strategies is,

$$V_{CIO} = v(1+k) \tag{6}$$

1.2 Opportunistic Mothers

When OMs interact with an IM or another OM they both act as independent caregivers. However, when the OM interacts with an CM there is a 1/2 chance the OM will reproduce first and gain the benefit of the allo-parent without reciprocating. The other 1/2 chance the CM will opt to reproduce first but the OM will refuse to allo-parent and both revert to acting as IMs. Thus for OMs the expected time to next birth becomes,

$$t_{ob} = qw + (1-p-q)w + \frac{1}{2}p\left(w + (w-s)\right) \tag{7}$$

where the expected offspring quality is,

$$(1-\frac{1}{2}p)v(1+k) + \frac{1}{2}pv(1+k+c) \tag{8}$$

put together gives the expected fitness of OMs,

$$W_{OM} = \left((1-\frac{1}{2}p)v(1+k) + \frac{1}{2}pv(1+k+c)\right)\sum_{i=0}^{\infty} u^{t_{ob}} \tag{9}$$

which simplifies to,

$$W_{OM} = \left((1-\frac{1}{2}p)v(1+k) + \frac{1}{2}pv(1+k+c)\right)\frac{1}{1-u^{t_{ob}}} \tag{10}$$

1.3 Independent Mothers

An Independent Mother's reproductive payoff is not contingent on any interactions with other females and is always,

$$W_{IM} = v(1+k)\frac{1}{1-u^{w}} \tag{11}$$

2 Paternal Interactions and Reproductive Success

The paternal contribution to infant care, k, differs depending on how the father of the offspring obtains resources. It takes on the value $k_{\bar{\mathrm{c}}}$ when females pair with an male who forages independently of other males and k_{C} when females pairs with a male who forms coalitions with other males, where $k_{\mathrm{C}} > k_{\bar{\mathrm{c}}}$. Then the fitness to the Coalition Male strategy, given the three types of female strategies in which the female partner is engaged, becomes,

$$W_{\mathrm{m}}(\mathrm{C}) = (1 - (xz_{\mathrm{C}} + (1-x)z_{\bar{\mathrm{c}}}))\,(m_c W_{CM}(k_C) + m_i W_{IM}(k_C) + m_o W_{OM}(k_{\mathrm{C}})) + z_{\mathrm{C}}\bar{W}_f$$

where $m_c = h + (1-h)p$, $m_i = (1-h)q$, and $m_o = (1-h)(1-p-q)$ are probabilities of interacting with the respective female strategies, adjusting for the level of positive assortment between male and female cooperative strategies (h). The first term accounts for the fitness gain from the offspring the male directly cares for with the female partner, while the second term accounts for fitness gains from extra-pair reproductive activity. Similarly, the fitness of the Non-coalition Male strategy is,

$$W_{\mathrm{m}}(\bar{\mathrm{c}}) = (1 - (xz_{\mathrm{C}} + (1-x)z_{\bar{\mathrm{c}}}))\,(pW_{CM}(k_{\bar{\mathrm{c}}}) + qW_{IM}(k_{\bar{\mathrm{c}}}) + (1-p-q)W_{OM}(k_{\bar{\mathrm{c}}})) + z_{\bar{\mathrm{c}}}\bar{W}_f$$
